# Amorphous Molybdenum Selenide Nanosheet as an Efficient Trap for the Permanent Sequestration of Vapor‐Phase Elemental Mercury

**DOI:** 10.1002/advs.201901410

**Published:** 2019-08-14

**Authors:** Zequn Yang, Hailong Li, Junwei Yang, Qin Yang, Jiexia Zhao, Jianping Yang, Wenqi Qu, Yong Feng, Kaimin Shih

**Affiliations:** ^1^ Department of Civil Engineering The University of Hong Kong Hong Kong SAR, China; ^2^ School of Energy Science and Engineering Central South University Changsha 410083 China; ^3^ College of Environmental Science and Engineering Nankai University Tianjin 300071 China

**Keywords:** amorphous MoSe_3_, elemental mercury, mercury selenide, nanosheets

## Abstract

The key challenge of vapor‐phase elemental mercury (Hg^0^) sequestration is the rational design of a sorbent with abundantly available ligands that exhibit excellent affinity toward Hg^0^ to simultaneously achieve a high uptake capacity and rapid capture rate. In this work, it is demonstrated how the correct combination of functional ligands and structural properties can form an ideal remediator for permanent Hg^0^ immobilization. The adsorption capacity of an amorphous molybdenum triselenide (MoSe_3_) nanosheet greater than 1000 mg g^−1^ is the highest recorded value compared to previously reported sorbents tested in a fixed‐bed reactor. Meanwhile, the uptake rate of 240 µg g^−1^ min^−1^ is also the highest recorded rate value. Mercury selenide as formed exhibits extremely low leachability when environmentally exposed. This impressive performance is primarily attributed to the appropriate layer space between the nanosheets that permeated Hg^0^ and the existence of diselenide (Se_2_^2−^) ligands that exhibit excellent affinity toward Hg^0^. Thus, this work not only provides a promising trap for permanent Hg^0^ sequestration from industrial and domestic sources with minimum hazard but also provides a detailed illustration of using structural advantages to obtain an ideal sorbent as well as guidance for the further development of Hg^0^ decontamination techniques.

## Graphical abstract

**An amorphous molybdenum selenide (MoSe_3_) sorbent** is developed for efficient and permanent vapor‐phase elemental mercury (Hg^0^) sequestration. After the rational design, the MoSe_3_ with increased interlayer space and abundant diselenide ligands shows the highest recorded performance for Hg^0^ capture. This work provides a new mindset for engineering a Hg^0^ trap using the appropriate combination of functional groups and structural properties.

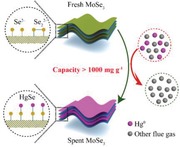

## 1 Introduction

Mercury (Hg), which is widely present in industrial flue gas and Hg‐laden consumables, poses tremendous environmental risks due to its high transportability, long‐distance persistence, and hypertoxicity.^1^ According to the newly released global mercury assessment, the annual emission of anthropogenic Hg to the immediate environment increased to 2500 tons in 2015 compared to 2000 tons in 2008.^2^ Among the various forms of Hg emitted, centralized control of elemental mercury (Hg^0^) faces the greatest challenge due to its volatility and insolubility, which leads to limitations of traditional air‐pollution control techniques for efficient degradation.^3^ For example, Hg emitted from industrial boilers/smelters generally includes Hg^0^, oxidized mercury (Hg^2+^) and particulate‐bound mercury (Hg^p^).^4^ Hg^2+^ and Hg^p^ can be efficiently captured by wet acid and particulate control facilities, while Hg^0^ persists in flue gas and acts as the main Hg form emitted to air.^5^ Thus, controlling Hg^0^ emissions is the key challenge for relieving the increasing mercury pollution worldwide.[qv: 2b]

The techniques used for Hg^0^ pollution remediation generally rely on sorbents with surface functional groups (including halogens, oxygen/oxides, sulfur/sulfide, etc.) that can accommodate Hg^0^.^6^ The activity, abundance, accessibility, and species of the functional groups coinfluence the Hg^0^ uptake capacity and rate. Moreover, the environmental stability of the final product is also an essential parameter to evaluate the feasibility of techniques. For a decent sorbent, it is important to take advantage of all of these requirements. For example, mineral sulfides (MSs) were recently found to very likely be potential sorbents for vapor‐phase Hg^0^ immobilization because they were capable of fully converting Hg^0^ into metabolically inactive and environmentally stable mercury sulfide (HgS).[qv: 6f] After adjusting the activity and accessibility of the sulfide species, molybdenum disulfide (MoS_2_) sheets exhibited an obvious enhanced Hg^0^ capture performance compared to bulky zinc monosulfide (ZnS).[qv: 6f,7] Recently, the uptake capacity was further increased using amorphous MoS_3_‐based sorbents with an abundant disulfide ligand (S_2_^2−^) that exhibited a higher affinity toward Hg^0^ than the monosulfide (S^2−^) in MoS_2_.^8^

Previous studies have strongly indicated that the rational design of an Hg^0^ remediator by combining different physical and chemical properties of the sorbent may derive an ideal material for permanent Hg^0^ sequestration. However, sorbents that include abundant functional groups exhibiting easy accessibility and high activity to simultaneously achieve a high capacity and rapid rate have rarely been realized. Even after the MSs were nanosized to achieve adequate exposure of the abundant sulfides (≥50% molar ratio), the Hg^0^ uptake capacity over the best MSs only reached less than 5% of its corresponding theoretical value, assuming that one mole of sulfide combines with one mole of Hg^0^.^4^ This insufficiency suggests that sulfide itself has limited affinity toward Hg^0^, and it is imperative to develop new functional groups to break through this barrier.

When searching for alternatives to the previously reported groups to significantly enhance the Hg^0^ uptake performance, it was observed that the binding affinity constant between Hg and selenium is 10^6^ times higher than the binding affinity constant between Hg and sulfur, but the solubility of mercury selenide (HgSe) is 10^3^‐fold lower.^9^ Selenide is the optimal natural ligand to combine with Hg to neutralize its neurotoxicity.^10^ These factors indicate not only that selenide‐based sorbents may exhibit a significantly improved adsorption performance compared to their sulfide counterparts but also that the obtained adsorbate (HgSe) will exhibit excellent environmental stability. To realize adequate selenide exposure, a nanosheet structure is preferential. Therefore, nanosheet‐structured molybdenum diselenide (MoSe_2_) is a promising candidate. However, MoSe_2_ has at least three drawbacks, i.e., the synthetic inconvenience of requiring a high temperature, relatively narrow layer spaces to exert its structural advantages to allow Hg^0^ in, and the lack of a diselenide ligand (Se_2_^2−^) that is likely to exhibit higher affinity to Hg^0^ than monoselenide (Se^2−^).^11^ Coincidentally, amorphous molybdenum selenides (MoSe*_a_*, *a*>2) generally contain rich Se_2_^2−^ ligands.^12^ Moreover, unlike MoS_3_, which is generally in an irregular phase,^13^ MoSe_a_ can be spontaneously solidified into a nanosheet at a relatively low temperature as it is the precursor for MoSe_2_ and shares a similar morphology with MoSe_2_.^14^ Based on this similarity, the increased surface area of MoSe_a_ compared to the surface area of MoSe_2_ 15 indicates that more active ligands would be exposed in MoSe_a_. Therefore, it is reasonable to speculate that MoSe_a_ would be a potential sorbent for Hg^0^ sequestration from industrial and domestic sources.

In this work, a rationally engineered amorphous MoSe_a_ was demonstrated to be an efficient trap for permanent vapor‐phase Hg^0^ sequestration due to its morphologic and structural properties. The as‐derived adsorption performance reached more than 1000 mg g^−1^, the highest recorded value compared to previously developed sorbents tested in fixed‐bed reactors. This work not only presents a potential alternative to traditional selections as a Hg^0^ remediator with minimum environmental effects but also provides guidance for the future design of effective Hg^0^ sorbents from a new perspective.

## 2 Results and Discussion

### 2.1 Structural and Morphologic Analyses

**Figure 1:**
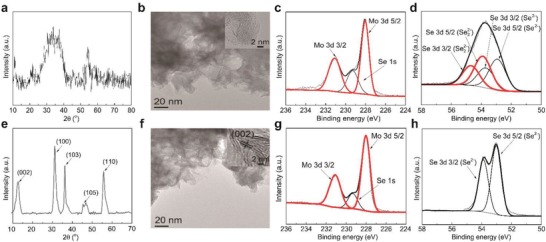
a–d) XRD, TEM (inserted with HRTEM), Mo 3d and Se 3d patterns of amorphous MoSe_3_; e–h) the corresponding counterparts of well‐crystallized MoSe_2_.

**Figure** **1**a presents the X‐ray diffraction (XRD) pattern of MoSe*_a_* as synthesized. Only two weak and broad peaks were observed at 25–40^o^ and 50–55^o^, which matches with the characteristic XRD peaks of amorphous MoSe_a_ reported in previous studies.^14^, ^15^ No well‐defined XRD peak was recoreded for MoSe*_a_* indicating its amorphism. The amorphous phase was further proven by high‐resolution transmission electronic microscopy (HRTEM) images showing that no characteristic lattice fringe was observed for MoSe*_a_*, while TEM images clarified that MoSe*_a_* existed in nanosheets (as shown in Figure 1b). This structure is in line with our proposal that MoSe*_a_*, as the precursor for MoSe_2_, shares similar structural properties with its crystallized phase. Moreover, to determine the valences of the molybdenum and selenide species in MoSe*_a_* and better predict its adsorption behavior and capacity, the X‐ray photoelectron spectroscopy (XPS) patterns of Mo 3d and Se 3d were recorded and are presented in Figure 1c,d, respectively. As shown, the Mo 3d envelope shows a doublet, with the Mo 3d 5/2 and 3d 3/2 peaks centering at 228.0 and 231.1 eV, respectively, which suggests that all molybdenum exists in Mo^4+^ in MoSe_a_, with negligible amounts of other valence states detected.^16^ For selenide, the Se 3d doublet of MoSe*_a_* at 53.1 and 54.0 eV is designated as the Se 3d 5/2 and 3d 3/2 characteristic peaks of monoselenide (Se^2−^), while the Se 3d peaks at 54.2 and 55.0 eV are due to the presence of diselenide (Se_2_^2−^).^12^ The XPS results demonstrate the copresence of Se^2−^ and Se_2_^2−^ in amorphous MoSe_a_ and that their molar ratio was nearly 1:1, giving a total charge of the Se species in MoSe_a_ of negative four (Se*_a_* ^4−^), which matches with the total charge of the molybdenum species (Mo^4+^). The presence of Se_2_^2−^ was expected to exhibit superior affinity toward Hg^0^ over Se^2−^, similar to S_2_^2−^ that is superior to S^2−^.^8^ In addition, the molar ratio of molybdenum and selenide in MoSe*_a_* was calculated based on the XPS results to be 1:3, which is helpful to derive its theoretical Hg^0^ adsorption capacity. Thus, amorphous MoSe*_a_* is denoted as MoSe_3_ in the following context.

For comparison, the characteristic counterparts of well‐crystallized MoSe_2_ are shown in Figure 1e–h. The characteristic peaks in the XRD results perfectly index to the (002), (100), (103), (105), and (110) crystal planes of MoSe_2_ (JCPD#29‐0914), which indicates that crystalline MoSe_2_ was successfully derived from amorphous MoSe_3_ (as shown in Figure 1a–d).^17^ The TEM image shows that as prepared MoSe_2_ exhibited an almost identical nanosheet structure with its precursor, while the HRTEM results indicate that the nanosheet was formed by the stacking of 3–4 layers in the <001> direction because the fringe of the side‐viewed nanosheet matches the lattice parameter between its (002) planes (0.68 nm) (as shown in Figure 1f).^18^ This assignment is reasonable based on the classic BFDH model, which relates the growth rate of specific crystal planes to their lattice fringes in an inverse proportion to self‐assemble into specific morphologies.^19^ In Figure 1g, it is shown that as in MoSe_3_, the molybdenum species in MoSe_2_ also presents as Mo^4+^, which is neutralized by 100% Se^2−^ anions instead of a 1:1 mixture of Se_2_^2−^ and Se^2−^ (as shown in Figure 1h).

The Brunauer–Emmett–Teller (BET) surface areas of MoSe_3_ and MoSe_2_ were 107.5 and 34.6 m^2^ g^−1^, respectively. Based on TEM images, MoSe_3_ and MoSe_2_ share almost identical morphologies with each other, strongly hinting that the formation of MoSe_2_ from MoSe_3_ may be achieved via an in situ crystallization process with the loss of 1/3 of selenide anions.^20^ Generally, an interlayer space larger than 0.6 nm is indispensable for nitrogen (van der Waals diameter of ≈0.3 nm) to permeate and form stable bimolecular layers to measure the areas of both the top and bottom surfaces.^21^ When the interlayer space is less than 0.3 nm, no N_2_ can penetrate; however, when the interlayer space is between 0.3 and 0.6 nm, only a monolayer of adsorbed N_2_ can be formed. For MoSe_2_, the interlayer space between the MoSe_2_–MoSe_2_ layers is only 0.36 nm (shown in Figure S1, Supporting Information). By contrast, the increased BET surface area of amorphous MoSe_3_ is probably attributed to its widened interlayer spaces (>0.6 nm), as the morphologies of the sorbents before and after annealing were negligibly changed. According to a previous study, an interlayer space of ≈0.3 nm was not adequate for Hg^0^ to form stable compounds, while an increased space of >0.6 nm was able to perform Hg^0^ chemisorption.[qv: 11b] Although the understanding of MoSe_3_ at the molecular level is still limited to date, its appropriate interlayer spaces may improve the accessibility of functional groups; hence, increasing its Hg^0^ sequestration performance.

### 2.2 Hg^0^ Uptake Capacity

**Figure 2:**
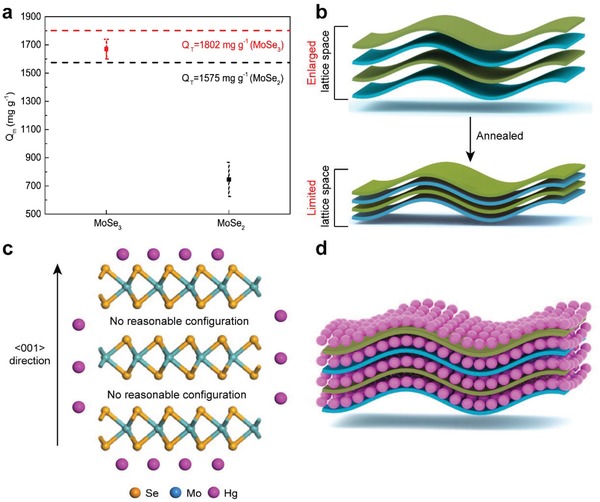
a) Adsorption capacity comparison between MoSe_3_ and MoSe_2_; b) proposed nanosheet structure of MoSe_3_ and MoSe_2_; c) DFT study of Hg^0^ adsorption over MoSe_2_; and d) assumed scenario for Hg^0^ adsorption over MoSe_3_.

As shown in **Figure** **2**a, amorphous MoSe_3_ exhibited a twofold higher maximum Hg^0^ adsorption capacity (*Q*_m_, 1670 mg g^−1^) than that of well‐crystallized MoSe_2_ (740 mg g^−1^), although their theoretical values (*Q*_T_) were not significantly different (as calculated by assuming that one mole of selenide can combine with one mole of Hg^0^). The *Q*_m_ of MoSe_3_ is equal to 93% of its corresponding *Q*_T_, while MoSe_2_ only performs at a 46% ratio, further supporting our above assumptions that amorphous MoSe_3_ has a lattice space suitable for Hg^0^ permeation (as shown in Figure 2b), because *Q*_m_ can only have such a close value to *Q*_T_ in MoSe_3_ if the selenides located in the interlayer spaces are adequately exploited.[qv: 11b] On the contrary, the *Q*_m_ of MoSe_2_ is inferior to the *Q*_m_ of MoSe_3,_ probably because the narrow interlayer spaces in MoSe_2_ did not allow Hg^0^ to permeate and form a stable configuration.

To further prove this point, a density functional theory (DFT) calculation was conducted for Hg^0^ adsorption over the MoSe_2_ surface and interlayer interval, for which all the stable configurations are shown in Figure S2 in the Supporting Information. The hollow sites were responsible for Hg^0^ immobilization over the MoSe_3_ (002) surface with a binding energy of −56.9 kJ mol^−1^ (listed in Table S1 in the Supporting Information), while Hg^0^ adsorption over the MoSe_3_ (100) surface had a binding energy of −125.8 kJ mol^−1^. This difference indicates that the (100) edges are preferential for Hg^0^ adsorption over MoSe_2_, which is generally in line with the results of a previous study.^22^ The Mulliken charge transfer from the adsorbent to adsorbed mercury further evidenced the superiority of the (100) edges (0.044 e) over the (002) surfaces (0.008 e) for anchoring mercury. However, no reasonable configuration can be derived (as shown in Figure 2c) after calculating the Hg^0^ capture between the interlayer spaces of MoSe_2_, which indicates that the active sites located in the space intervals are inaccessible to Hg^0^. This inaccessibility primarily caused the *Q*_m_ of MoSe_2_ to be significantly inferior to its corresponding *Q*_T_. On the contrary, although the molecular structure of MoSe_3_ remains unknown, the *Q*_m_ of MoSe_3_ accounts for nearly 100% of its *Q*_T_, indicating the interlayer spaces of amorphous MoSe_3_ are available for Hg^0^ immobilization (as shown in Figure 2d). This assumption is generally in line with our assumptions regarding the interlayer spaces derived from the BET surface area results.

### 2.3 Hg^0^ Uptake Rate

**Figure 3:**
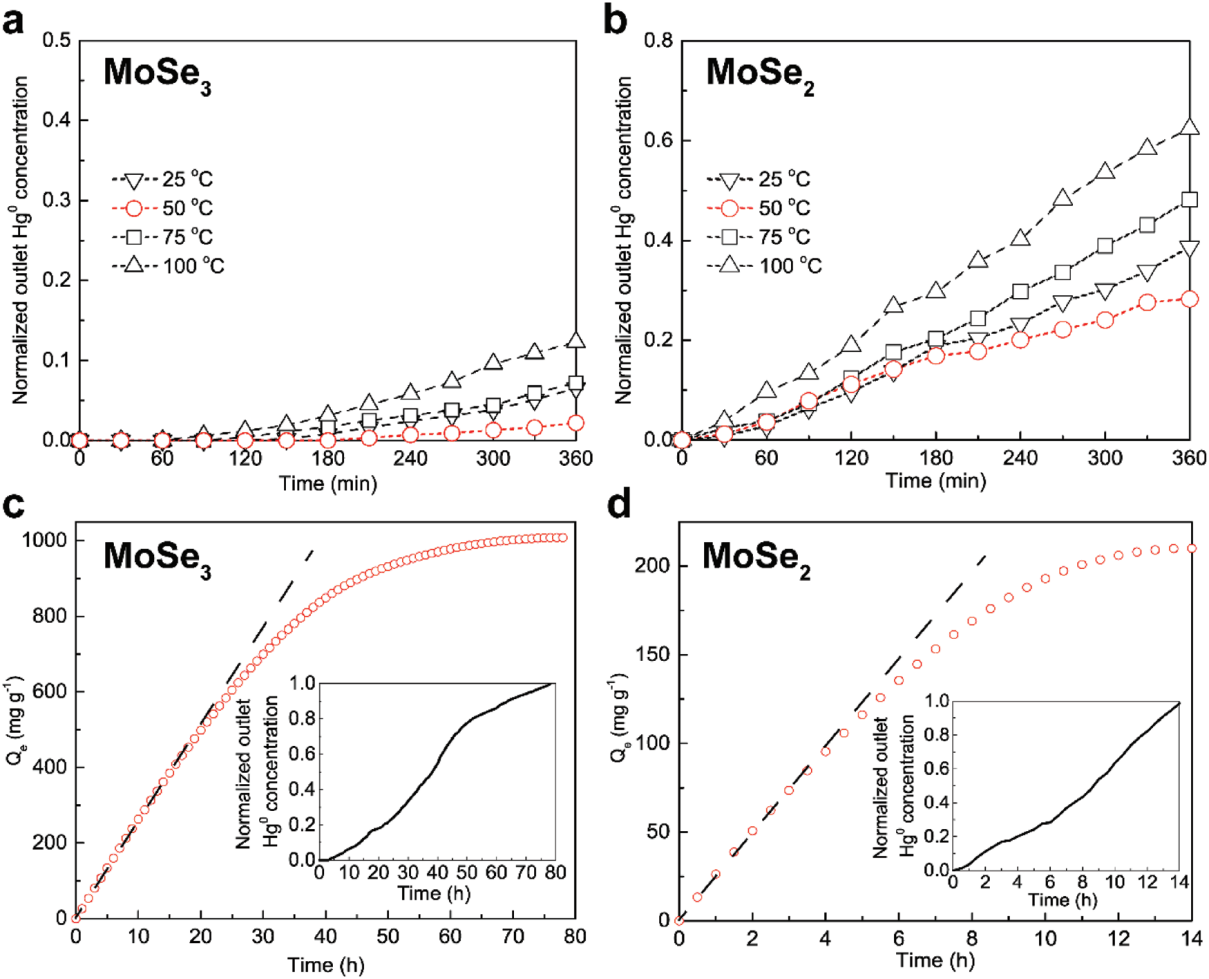
a,b) Influence of the reaction temperature on the Hg^0^ capture rate over MoSe_3_ and MoSe_2_; c,d) the corresponding equilibrium capacities (*Q*_e_) of MoSe_3_ and MoSe_2_ at their optimal temperature (inserted with the breakthrough curves).

As shown in **Figure** **3**a, MoSe_3_ exhibited an normalized outlet Hg^0^ concentration (to the inlet Hg^0^) concentration of less than 0.15, regardless of temperature, during the 6 h experiments. Only 1 mg of sorbent was used with a gas hourly space velocity (GHSV) of 7200 000 h^−1^, which is thousands of times higher than under real‐world conditions. These results indicate the superior fast kinetics of Hg^0^ adsorption over MoSe_3_. The optimal reaction temperature of 50 °C was probably because a relatively high temperature provided the reaction with more activation energy.^23^ However, further increasing the temperature would lead to the possible decomposition of the adsorbate, i.e., HgSe, and adversely influence Hg^0^ sequestration.^24^ The same trend was observed for Hg^0^ adsorption over crystalline MoSe_2_ (as shown in Figure 3b). However, the normalized outlet Hg^0^ concentration reached as high as 0.39, 0.28, 0.48 and 0.62 at 25, 50, 75, and 100 °C. These values are suppressed by the performance by MoSe_3_. The optimal temperature of 50 °C was selected to derive the breakthrough curves to further investigate the Hg^0^ adsorption rate over the as‐synthesized sorbents.

**Figure 4:**
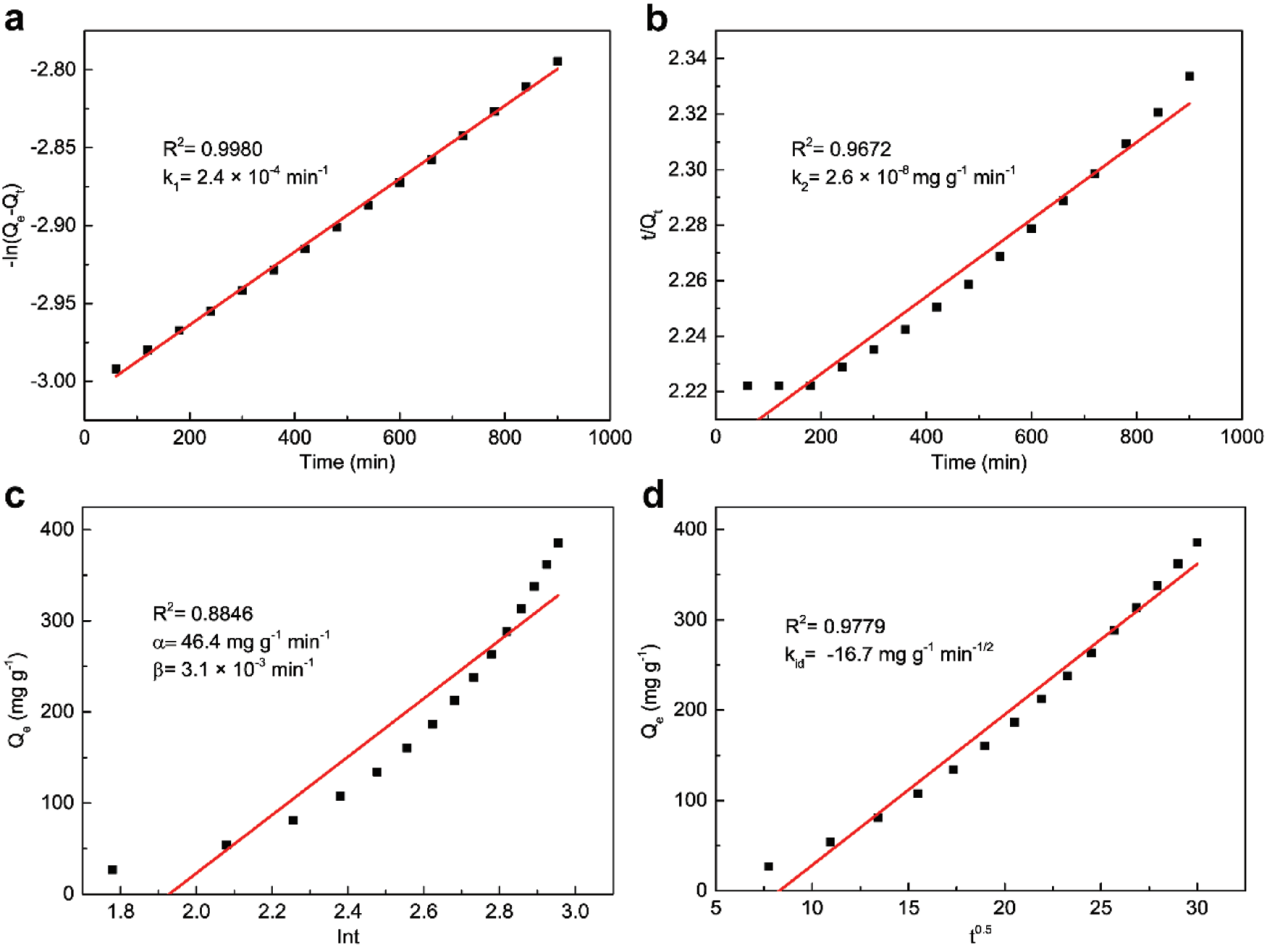
Hg^0^ adsorption rate of MoSe_3_ simulated by a) pseudo‐first‐order, b) pseudo‐second‐oder, c) Elovich, and d) intraparticle diffusion models.

As shown in Figure 3c,d, *Q_e_* at 50 °C of MoSe_3_ and MoSe_2_ reached more than 1000 and 200 mg g^−1^, respectively, under a 1.5 mg m^−3^ of Hg^0^ feed. For MoSe_3_, nearly 80 h was needed for the adsorption rate to equal the desorption rate (equilibrium state), which was 5.5 times longer than the time required for MoSe_2_. From the slope of the breakthrough curves (marked as dashed lines in Figure 3c,d), it is clear that the initial Hg^0^ adsorption rate of MoSe_3_ is obviously faster than the initial Hg^0^ adsorption rate of MoSe_2_, primarily due to the adsorption sites in amorphous MoSe_3_ exhibiting higher affinity toward Hg^0^ than the adsorption sites in MoSe_2_ as the abundances of the sites are both adequate at the initial stage in an open space where the Hg^0^ concentration is not high enough. The specific initial Hg^0^ capture rate of MoSe_3_ was determined by simulations using different kinetic models (as shown in **Figure** **4**a–d).^25^ As shown, the pseudo‐first‐order, pseudo‐second‐order, Elovich, and Intraparticle diffusion models derived correlation coefficients (*R* ^2^) of 0.9980, 0.9672, 0.8846, and 0.9779, respectively. Except for the pseudo‐first‐order kinetic model, the simulations by the other three models induced relatively high deviations from the experimental data. The Hg^0^ adsorption rate (*R*_a_) as calculated by pseudo‐first‐order kinetics was 240 µg g^−1^ min^−1^.

**Figure 5:**
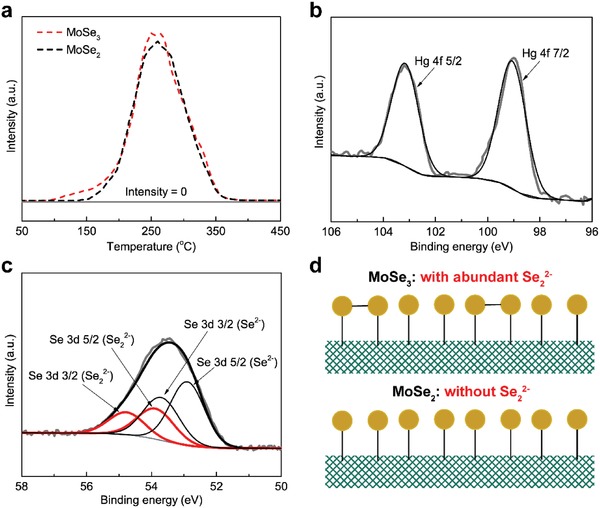
a) Hg‐TPD patterns of MoSe_3_ and MoSe_2_; b,c) Hg 4f and Se 3d patterns of spent MoSe_3_; and d) diagrammatic illustration of the selenide species difference between MoSe_3_ and MoSe_2_.

To provide evidence that Se_2_^2−^ accounts for the increased Hg^0^ uptake rate of MoSe_3_ compared to MoSe_2_, Hg‐TPD was conducted to determine the adsorbate species on MoSe_3_ and MoSe_2_ (as shown in **Figure** **5**a). As shown, the characteristic desorption/decomposition peaks for Hg‐laden MoSe_3_ and MoSe_2_ centered at ≈260 °C, both of which were due to the existence of HgSe with no other mercury‐related species detected.^24^ The Hg 4f 7/2 and 5/2 doublets located at 99.5 and 103.2 eV were indicative of spent MoSe_3_, which matched the binding energy of the Hg–Se bond, further proving that the mercury in MoSe_3_ exists as HgSe (as shown in Figure 5b).^26^ Moreover, as shown in Figure 5c, Se_2_^2−^ significantly decreased in spent MoSe_3_ with the increase of Se^2−^ species, suggesting that the Hg^0^ removal over MoSe_3_ can be expressed by the following reaction at the initial stage

(1) $${\mathrm{Hg}}^{0}+{\mathrm{Se}}_{2}^{2-}\to \mathrm{HgSe}+{\mathrm{Se}}^{2-}$$

On the contrary, for MoSe_2_, due to absence of Se_2_^2−^ ligands (as shown in Figure 5d), Se^2−^ provides the only possible active sites for Hg^0^ accommodation to form HgSe^24^

(2) $${\mathrm{2Hg}}^{0}+{\mathrm{MoSe}}_{2}\to \mathrm{2HgSe}+{\mathrm{Mo}}^{0}$$

Thus, it is reasonable to attribute the greatly increased Hg^0^ adsorption rate of MoSe_3_ to the existence of Se_2_^2−^ chelating sites with high affinity to Hg^0^.

### 2.4 Comparison with Previously Reported Sorbents

**Figure 6:**
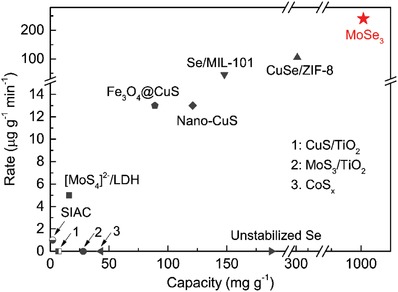
Equilibrium adsorption capacity and adsorption rate comparison between MoSe_3_ and previously reported sorbents for Hg^0^ sequestration.

The *Q_e_* and *R_a_* of MoSe_3_ were compared with those of previously reported Hg^0^ sorbents tested under similar conditions, and the full results are listed in Table S2 in the Supporting Information. Moreover, **Figure** **6** shows some typical sorbents at their optimal temperatures to provide a clearer comparison, where a rate equal to zero indicates that the adsorption rate was not reported. As shown in Table S2 in the Supporting Information and Figure 6, the rationally designed MoSe_3_ outperformed all the previously reported sorbents for Hg^0^ sequestration and had a relatively decent performance.^4^, ^5^, ^7^, ^23^, ^24^, ^27^ Specifically, the adsorption capacity of MoSe_3_ was at least 500, 10, and 5 times greater than the adsorption capacities of carbon‐, sulfide‐, and selenium‐based materials, respectively, while its adsorption rate was at least one order of magnitude higher. Compared to a copper selenide (CuSe)‐based sorbent, the adsorption capacity of MoSe_3_ was shown to be threefold greater, and its adsorption rate was improved by twofold. Even at 75 and 100 °C that are not the optimal operation temperature for MoSe_3_, MoSe_3_ still exhibited the highest adsorption capacities compared to the capacities of previously reported sorbents at their optimal temperatures (as shown in Table S2 and Figure S3, Supporting Information). These improvements are mainly attributed to the rational design because: 1) the affinity between selenide/selenium and Hg^0^ is superior to the affinity between sulfide/sulfur and Hg^0^ and (2) the abundance of diselenide and its corresponding accessibility are significantly enhanced in MoSe_3_ compared to other selenide‐/selenium‐based sorbents. The excellent performance of MoSe_3_ for Hg^0^ sequestration indicates its great potential to be widely applied under real‐world conditions.

### 2.5 Implications for Real‐World Applications

**Figure 7:**
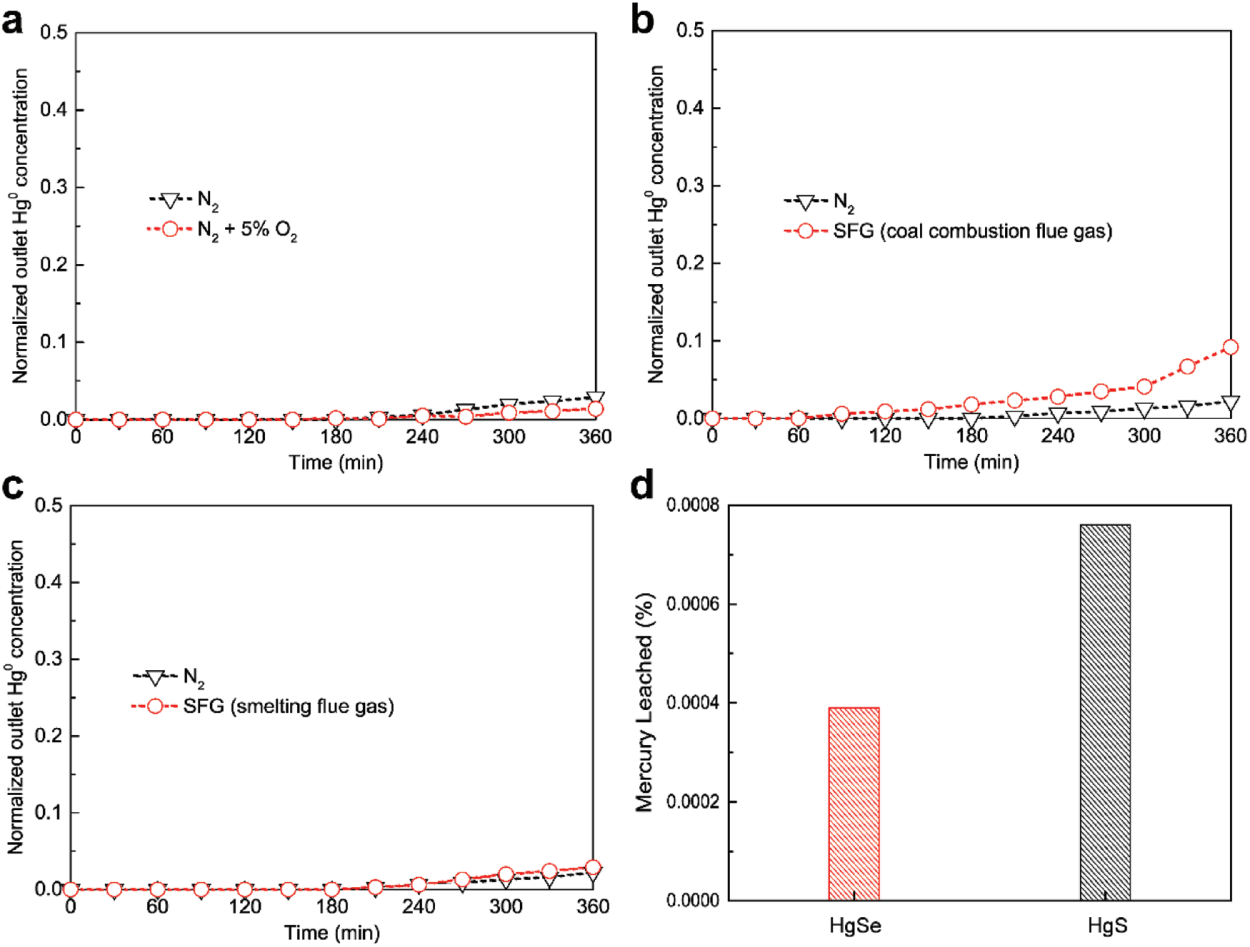
a–c) Hg^0^ removal under N_2_ + 5% O_2_, SFG (coal combustion flue gas) and SFG (smelting flue gas), respectively; d) Hg^0^ leaching from HgSe‐ and HgS‐laden sorbents.

To quantitatively determine the potential of MoSe_3_ for Hg^0^ adsorption, three typical flue gas atmospheres were chosen to simulate the real‐world conditions, i.e., N_2_ plus O_2_, simulated flue gas (SFG) from coal combustion flue gas and SFG from smelting flue gas. As shown in **Figure** **7**a, the presence of oxygen (O_2_) had a negligible influence on Hg^0^ capture over MoSe_3_, which indicates that MoSe_3_ exhibits excellent resistance to oxidation at relatively low temperatures (50 °C). Its decent resistance toward oxidation also indicates that MoSe_3_ may be a promising trap for Hg^0^ decontamination from domestic sources.

As shown in Figure 7b, the Hg^0^ removal performance was slightly inhibited by coal combustion flue gas, possibly because: (1) the presence of water vapor (H_2_O) competed with Hg^0^ for active sites[qv: 27k] and/or (2) the addition of nitrogen monoxide (NO) plus O_2_ oxidized the active selenide sites into inert selenite species.^28^ An injection strategy is generally adopted for Hg^0^ capture from coal combustion flue gas. In an injection scenario, the sorbent is injected before the air pollution control devices with a residence time of less than 30 min.^28^ Thus, a 30 min timespan is the most critical parameter to evaluate the suitability of sorbents to be applied to treat coal combustion flue gas. Considering this prerequisite, MoSe_3_ is still a potential sorbent for Hg^0^ capture from coal combustion flue gas because the Hg^0^ removal performance over MoSe_3_ was negligibly influenced by simulated SFG within 30 min with an extremely high GHSV of 7200 000 h^−1^.

For simulated smelting flue gas, a high concentration of SO_2_ also had a negligible detrimental effect on Hg^0^ sequestration (as shown in Figure 7c). This property distinguished the MoSe_3_ sorbent from the traditional Boliden–Norzink process adopting mercurous chloride (HgCl_2_) as the adsorption liquor to transform vapor‐phase Hg^0^ into Hg_2_Cl_2_ solid, the performance of which is significantly inhibited by the presence of abundant SO_2_. This inhibitive effect is primarily attributed to SO_2_ being able to reduce HgCl_2_ and introduce redundant Hg^0^ into the flue gas.^29^ Moreover, the adsorbent and adsorbate in the Boliden–Norzink process are both hypertoxic^30^ and are preferentially replaced by harmless selenide compounds. Thus, it is proper to conclude that MoSe_3_ is also an ideal alternative to HgCl_2_ for Hg^0^ remediation in smelting flue gas.

More importantly, the as‐formed HgSe over the MoSe_3_ surface is ultrastable when environmentally exposed, which is manifested by its extremely low leachability (as shown in Table S3 in the Supporting Information and Figure 7d). The leaching ratio of HgSe is even lower than the leaching ratio of its sulfide counterpart (HgS), which is renowned as the most stable form of naturally enriched mercury ore.^24^ The mercury concentration in leachate of 0.36 µg L^−1^ is far below the safe limit (200 µg L^−1^) mandated by the United States Environmental Protection Agency (EPA) for classifying a material as hazardous waste.^31^ Moreover, the extremely low leaching of mercury from the MoSe_3_ surface even meets the upper mercury content limit in drinking water (2.0 µg L^−1^) as imposed by the EPA,^32^ suggesting that Hg‐laden HgSe can be directly dumped and landfilled with a minimum reemission risk. Vapor‐phase Hg^0^ can be permanently sequestrated from a hypertoxic and volatile form into the most inert and stable form (HgSe) on MoSe_3_.

## 3 Conclusions

To realize a simultaneous high capacity and rapid rate of Hg^0^ capture from various sources, the amorphous MoSe_3_ nanosheet synthesized by a one‐step hydrothermal method was used for the first time for permanent Hg^0^ sequestration. Compared to the well‐crystallized MoSe_2_ nanosheet, MoSe_3_ performed better primarily because of its abundant and accessible Se_2_^2−^ ligands, which exhibited excellent affinity toward Hg^0^. The adsorption capacity reached more than 1000 mg g^−1^, which is the highest recorded value compared to previously reported sorbents tested in a fixed‐bed reactor. The uptake rate reached high as 240 µg g^−1^ min^−1^ and was enhanced compared to traditional Hg^0^ traps by orders of magnitude. Moreover, the MoSe_3_ nanosheet showed excellent resistance to complicated gas atmospheres from various industrial/domestic sources, and the as‐formed adsorbate HgSe exhibited extremely low leachability when environmentally exposed. This work not only proposes an ideal sorbent for Hg^0^ decontamination from variable industrial and domestic sources but also provides guidance for the future design of efficient sorbents with a new mindset of morphologic and structural optimization.

## 4 Experimental Section

*Sorbent Preparation*: MoSe_3_ was prepared using a one‐step hydrothermal method. In a typical procedure, 2 mmol of sodium molybdate (Na_2_MoO_4_, Sinopharm) was dissolved in deionized water. In a separate beaker, 6 mmol of selenium powder (Se, Aladdin) was added to 15 mL of a hydrazine hydrate solution (N_2_H_4_·H_2_O, 80%, Sinopharm) and stirred for at least one day. Then, the Na_2_MoO_4_ solution was poured into the Se‐N_2_H_4_·H_2_O mixture, which was transferred into a Teflon‐lined autoclave and heated in an oven at 180 °C for 12 h. The as‐obtained black solid was separated by filtration, washed with deionized water and ethanol several times, and dried at 70 °C under vacuum for one night to produce amorphous MoSe_3_. For comparison, well‐crystallized MoSe_2_ was synthesized by annealing MoSe_3_ at 450 °C under pure argon (Ar) for 10 h.

*Sorbent Characterization*: The crystallinity of the sorbents was measured by X‐ray diffraction (XRD, D8 Bruker AXS, Germany) with two theta from 10° to 80° in Cu_α_ (λ = 0.154 nm) radiation. A transmission electron microscope (JEOL 2100F, Japan) was used to determine the morphologies of the as‐prepared sorbents. The fresh and spent sorbents were characterized by their X‐ray photoelectron spectroscopy (XPS, Thermo ESCALAB 250Xi) spectra with a C 1s binding energy value of 284.8 eV as the reference. Spent MoSe_3_ was obtained by pretreating fresh MoSe_3_ under 1.5 mg m^−3^ Hg^0^ for 120 h. The Brunauer–Emmett–Teller surface area of the sorbents was determined by the N_2_ adsorption and desorption method with a BET analyzer (ASAP 2020, Micromeritics, USA). Before BET testing, the prepared sorbents were purged in pure N_2_ for 4 h to obtain a clean surface.

*Determination of the Hg^0^ Adsorption Capacity and Rate*: The maximum Hg^0^ adsorption capacities of MoSe_3_ and MoSe_2_ were determined by a nested tube reactor (as shown in Figure S4, Supporting Information). The Hg^0^ source was placed in the bottom of the inner reactor without a lid, while the sorbent was settled on the top of the inner reactor and separated by a filter paper that was capable of permeating Hg^0^ but inert for Hg^0^ adsorption. Before the tests began, the sorbents without and with filter paper, which were denoted as *W*_sorbent_ and *W*_in_, respectively, were both weighed. Then, the Hg^0^ source in the sealed tube reactor was heated by an oil bath at 140 °C and held for 1 d. The Hg^0^ concentration reached a nearly infinite value in the sealed nested reactor and attacked every possible adsorption site on MoSe_3_ or MoSe_2_. Then, the filter paper with sorbents was taken out, and its weight (*W*_out_) was determined again. The maximum Hg^0^ uptake capacity (*Q*_m_, mg g^−1^) of the sorbents was calculated by Equation (3)

(3) $$Q_{m}=\frac{W_{\mathrm{out}}-W_{\mathrm{in}}}{W_{\mathrm{sorbent}}}$$

As the Hg^0^ uptake rate must be derived by fitting breakthrough curves and requires in situ Hg^0^ concentration detection, a fixed‐bed reactor was used to test Hg^0^ capture (as shown in Figure S5, Supporting Information) under pure N_2_. Under this condition, the temperature affected the Hg^0^ adsorption behavior since the temperature significantly affected the site activity (adsorption rate) and desorption behavior (desorption rate).^24^ When the adsorption rate equaled the desorption rate, the sorbent was penetrated and reached an equilibrium adsorption capacity (*Q*_e_) with *Q*_e_ < *Q*_m_. Thus, investigating the impact of temperature was the first step to properly define the corresponding adsorption rate. In the fixed‐bed reaction system, the total flow rate was precisely controlled by mass flow controllers (MFCs) to be 300 mL min^−1^. A Dynacal Hg^0^ permeation device (VICI Metronics) was used to produce a constant feed of Hg^0^ vapor. A fixed‐bed reactor made of Pyrex with an inner diameter of 1 cm was placed into a tubular furnace equipped with a temperature adjustment system to control the reaction temperature. The concentration of Hg^0^ was detected with a mercury analyzer (VM3000, Mercury Instrument, Inc.) and continuously recorded by a connected computer. Before each test, the Hg^0^ carried by different carrier gases bypassed the reactor and was used to detect the inlet Hg^0^ concentration (*C*_in_). Until the fluctuation of *C*_in_ was less than 10 µg m^−3^ within 30 min, Hg^0^ could pass through the sorbent. The as‐recorded Hg^0^ concentration was denoted as the outlet Hg^0^ concentration (*C*_out_). Hence, the real‐time Hg^0^ adsorption capacity (*Q*_t_, mg g^−1^) was calculated by Equation (2)

(4) $$Q_{t}=\frac{1}{m}\int_{t_{1}}^{t_{2}}\left(C_{\mathrm{in}}-C_{\mathrm{out}}\right)\times f\times dt$$

where *f* (m^3^ min^−1^) is the gas flow rate, *m* (g) is the mass of the sorbent, and *t* (min) is the adsorption process duration time. To obtain the equilibrium Hg^0^ capture capacity (*Q*_e_) in this case, *t*_1_ and *t*_2_ were denoted as zero and the time *C*_in_ = *C*_out_ (equilibrium time), respectively. Despite a relatively high *C*_in_ slightly enhances the *Q*_e_,^24^ appropriate *C*_in_ is also required for sorbents with different *Q*_m_ to limit the total experimental time and avoid the flucturation of *C*_in_. Based on the preliminary experiments, a *C*_in_ of 1.5 mg m^−3^ was used to significantly decrease the experimental errors resulted from dramatic *C*_in_ fluctuation in a lengthy test.

Then, the Hg^0^ adsorption rate of MoSe_3_ was obtained by simulation with different kinetic models, i.e., a pseudo‐first‐order model, pseudo‐second‐order model, Elovich model, and Intra‐particle diffusion model. The optimal model with the highest coefficient factor (*R* ^2^) was chosen to describe the kinetic behavior of Hg^0^ adsorption on the MoSe_3_ surface.

1) Pseudo‐first‐order model

The pseudo‐first‐order kinetic model was based on the mass balance. The Hg adsorption rate was proportional to the difference between the equilibrium capacity and the adsorbed amount at any time, as described as follows

(5) $$\frac{dQ_{t}}{dt}=K_{1}\left(Q_{e}-Q_{t}\right)$$

Equation (3) could be modified to the following equation based on the initial conditions of *t* = 0, *Q*_t_ = 0

(6) $$Q_{t}=Q_{e}\left(1-e^{-k_{1}t}\right)$$

where the pseudo‐first‐order kinetic constant (*k*_1_, min^−1^) can be determined by fitting the adsorption breakthrough curve.

2) Pseudo‐second‐order model

The pseudo‐second‐order model considers that the surface diffusivity is inversely proportional to the square of the concentration variation on the sorbent surface, which could be described by Equation (5)

(7) $$\frac{dQ_{t}}{dt}=k_{2}{\left(Q_{e}-Q_{t}\right)}^{2}$$

Equation (5) could be modified to the following equation based on the initial conditions of *t* = 0, *Q_t_* = 0

(8) $$\frac{t}{Q_{t}}=\frac{1}{k_{2}Q_{e}^{2}}+\frac{1}{Q_{e}}t$$

where the pseudo‐second‐order kinetic constant (*k*_2_, mg g^−1^ min^−1^) can be determined by fitting the adsorption breakthrough curve.

3) Elovich model

The Elovich model assumes that sorption takes place in two phases: (1) a fast initial reaction associated with the movement of the sorbate to external sites and (2) a slower diffusion in and out of the micropores over the sorbent. This model can be described by the following equation

(9) $$\frac{dQ_{t}}{dt}=\alpha e^{-\beta Q_{t}}$$

where α (mg g^−1^ min^−1^) represents the initial rate and β (min^−1^) is related to the extent of surface coverage and the activation energy for chemisorption. This equation can be modified into

(10) $$Q_{t}=\frac{1}{\beta }\ln(\alpha \beta )\;\;+\;\;\frac{1}{\beta }\ln t$$

4) Intra‐particle diffusion model

The intraparticle diffusion model assumes that the intraparticle diffusivity is constant and that the diffusion direction is radial. The model can be interpreted by the following equation

(11) $$Q_{t}=k_{\mathrm{id}}t^{0.5}+C$$

where *k*_id_ (mg g^−1^ min^−1/2^) represents the intraparticle diffusion rate constant and *C* (mg g^−1^) is proportional to the boundary layer.

*First Principle Calculation*: Please refer to the Supporting Information, First Principle Calculation section.

*Mercury Temperature Programmed Desorption/Decomposition (Hg‐TPD)*: To identify the mercury species adsorbed on the surface of the sorbent, mercury temperature‐programmed desorption (Hg‐TPD) tests were conducted. Before the Hg‐TPD tests, MoSe_3_ and MoSe_2_ were pretreated with 1.5 mg m^−3^ Hg^0^ balanced in N_2_ at 50 °C for 30 min to assure that enough mercury was accumulated on the surface of the sorbent. Then, the Hg^0^ feed was cut off, and the Hg‐laden sorbents were purged by pure N_2_ at 50 °C until the outlet Hg^0^ concentration stably equaled zero. The Hg‐TPD tests were conducted from 50 to 450 °C with a heating rate of 10 °C min^−1^.

*Hg^0^ Adsorption Activity Test under Simulated Real‐World Conditions*: For Hg^0^ removal under real‐world conditions, Hg^0^ generally exists in an open instead of a closed space, and the Hg^0^ adsorption capacity cannot reach its maximum value. Thus, the fixed‐bed reactor was selected to simulate the real‐world conditions in which the Hg^0^ feed continuously flows through the sorbents.^33^ Three typical gas atmospheres, i.e., N_2_ plus 5% oxygen (O_2_), coal combustion flue gas, and nonferrous smelting flue gas, were adopted. The coal combustion flue gas contained 5% O_2_, 100 ppm nitrogen monoxide (NO), 300 ppm sulfur dioxide (SO_2_), 12% carbon dioxide (CO_2_), and 8% water vapor (H_2_O) carried by N_2_, and the smelting flue gas comprised 5% O_2_ plus 1.5% SO_2_. The different flue gas components were supplied by compressed gas cylinders containing N_2_, O_2_, SO_2_, CO_2_, and NO. Water vapor (H_2_O) was introduced into the reactor by a separate flow of N_2_. The total flow rate was precisely controlled by MFCs. Sorbent dosages of 1 mg were adopted in all experiments, which is much lower than a real‐world situation to avoid the possible influence of flue gas components being concealed by excessive sorbent used.

*Mercury Leaching Test*: Please refer to the Supporting Information, Mercury Leaching Test section.

## Conflict of Interest

The authors declare no conflict of interest.

## Supporting information

Supplementary
