# Supplementary material for: Amorphous Molybdenum Selenide Nanosheet as an Efficient Trap for the Permanent Sequestration of Vapor‐Phase Elemental Mercury
Source: Adv Sci (Weinh). 2019 Aug 14;6(20):1901410. doi: 10.1002/advs.201901410 (PMC6794631; doi:10.1002/advs.201901410)

Copyright WILEY-VCH Verlag GmbH & Co. KGaA, 69469 Weinheim, Germany, 2019.

ADVANCED SCIENCE
Open Access

Supporting Information

for *Adv. Sci.,* DOI: 10.1002/advs.201901410

Amorphous Molybdenum Selenide Nanosheet as an Efficient Trap for the Permanent Sequestration of Vapor-Phase Elemental Mercury

Zequn Yang, Hailong Li, Junwei Yang, Qin Yang, Jiexia Zhao, Jianping Yang, Wenqi Qu, Yong Feng, and Kaimin Shih**

Copyright WILEY-VCH Verlag GmbH & Co. KGaA, 69469 Weinheim, Germany, 2019.

Supporting Information

Amorphous Molybdenum Selenide Nanosheet as an Efficient Trap for the Permanent Sequestration of Vapor-Phase Elemental Mercury

Zequn Yang[a], Hailong Li[b], Junwei Yang[c], Qin Yang[b], Jiexia Zhao[b], Jianping Yang[b], Wenqi Qu[b], Yong Feng[a], Kaimin Shih[a**]*

a. Department of Civil Engineering, The University of Hong Kong, Hong Kong SAR, China

b. School of Energy Science and Engineering, Central South University, Changsha, 410083, China

c. College of Environmental Science and Engineering, Nankai University, Tianjin, 300071, China

Revision submitted to **Advanced Science**

*To whom correspondence should be addressed:

TEL: +86-18670016725

E-mail: hailongli18@gmail.com

**To whom correspondence should be addressed:

TEL: +852-2859-2973

Email: kshih@hku.hk

First principle calculation

The $MoSe_2$ model was in hexagonal structure with space group P63/mmc (a=b=3.288 Å, c=12.900 Å, α=β =90°, γ=120°). A 9-layer slab with a (3×3) or (3×1) unit cell was used to model the structure of $MoSe_2$(002) or (100) surface. A 15 Å vacuum region between the slabs was constructed to avoid the spurious interactions. All calculations were conducted with the quantum mechanics based $Dmol^3$ program package in Materials Studio 8.0. The exchange correlation potential was determined by Perdew-Burke-Ernzerhoff (PBE) approximation in the place of the generalized gradient approximation (GGA) scheme. The interaction between valance electrons, inner electrons and atomic nucleus was performed with the double numerical basis sets plus polarization functional (DNP). The Monkhorst-Pack scheme k-points grid of 1×1×1 was used to simplify the Brillouin zone and the real space basis set functions are adjusted to be 4.4 Å. The DFT-D method of Grimme was adopted to consider the van der waals forces between the layers. The criteria for the tolerances of energy, force, displacement, and SCF convergence criteria are set as 10^{-5} Ha, 4×10^{-3} Ha $Å^{-1}$, 5×10^{-3} Å, and 10^{-5}, respectively. A Methfessel-Paxton smearing of 0.005 Ha was used to improve calculation performance. Gas-phase species (Hg^0) molecule was also optimized separately in a large crystal cell of 10×10×10 Å.

The binding energy (kJ/mol) of mercury species over $MoSe_2$ crystalline was determined by the following equation:

$$BE = E_{sorbent\text{-}adsorbate} - (E_{sorbent} + E_{adsorbate}) \quad (1)$$

where the $E_{sorbent\text{-}adsorbate}$, $E_{sorbent}$ and $E_{adsorbate}$ (kJ/mol) represent the total energy of the sorbent and adsorbate system, sorbent with clean surface and isolated adsorbate molecule, respectively. A more negative binding energy points to a higher capability of the adsorbate to be adsorbed on the sorbent surface, and a positive binding energy means that the adsorbate cannot be immobilized on the sorbent.

Mercury leaching test

A modified toxicity characteristic leaching procedure (TCLP) method was applied to simulate the leachability of mercury when the $MoSe_3$ was disposed in landfills. 0.2 g of $MoSe_3$ was pretreated at 50 ^{o}C by 1.5 mg m^{-3} for 4 h to be Hg-laden $MoSe_3$. 5.7 ml of glacial acetic acid was diluted by deionized water to 1 L to be the leachate. The Hg-laden $MoSe_3$ was placed in an extraction bottle containing 4 g of the leachate and kept shaking by a rotary agitation apparatus for 18 h. Then, the solution in the extraction bottle was collected and the contained mercury concentration was determined by an atomic fluorescence spectrometer (AFS, Kechuang Haiguang Instruments Co. Ltd., China).

List of Tables:

Table S1. Parameters for Hg^0 adsorptions on $MoSe_2$ (002) and (100) surfaces.

Table S2. Hg^0 adsorption performance comparison.

Table S3. Leachability of mercury from Hg-laden selenide and sulfide.

Table S1. Parameters for Hg^0 adsorptions on $MoSe_2$ (002) and (100) surfaces.

Configurations	Binding energy (kJ mol^{-1})	Mulliken charge of Hg
Hg adsorbed on (002) surface	-56.9	0.008
Hg adsorbed on (100) surface	-125.8	0.044

Table S2. Hg^0 adsorption performance comparison.

Sorbents	Inlet Hg^0 concentration ($\mu g\ m^{-3}$)	Reaction temperature (^oC)	Capacities ($mg\ g^{-1}$)	Rates ($\mu g\ g^{-1}\ min^{-1}$)	Reference
$MoSe_3$	1500	50	1008	240	This work
$MoSe_3$	1500	75	830	-	This work
$MoSe_3$	1500	100	514	-	This work
$MoSe_2$	1500	50	210	-	This work
CuSe/ZIF-8	200	50	305	105	24
Unstabilized Se	60	25	188	-	27o
Se/MIL-101	1000	100	148	45	5
$MoS_2/\gamma\text{-}Al_2O_3$	30	50	19	0.1	7
$[MoS_4]^{2-}$/CoFe-LDH	350	75	16	5	27n
S/FeS_2	70	60	3	-	23
CoS_x	1300	100	43	-	27m
Nano-sized CuS	90	75	121	13	4
Fe_3O_4@CuS	90	75	89	13	27l
Fe_xS	120	60	0.2	0.3	27k
H_2S modified ilmenite	110	60	0.2	0.5	27j
H_2S modified Fe-Ti spinel	110	60	0.7	2	27i
CuS/TiO_2	4300	100	7	-	27g
MoS_3/TiO_2	4400	80	28	-	8
$(Fe_{3-x}Mn_x)_{1-\sigma}O_4$	1000	250	5	-	27e

Polysulfide enriched polymer	-	60	0.6	-	27c
Sulfur impregnated activated carbon	25	120	2	1	27a
Bare activated carbon	4900	140	0.4	-	27b

Table S3. Leachability of mercury from Hg-laden selenide and sulfide

	Initial Hg content in sorbent (μg/g)	Hg concentration in leachate (μg/L)	Hg leached (%)	Reference
0.8NC-ZIF	540.0	0.36	0.00039	This work
S/FeS_2	363.6	0.70	0.00076	23

List of Figures:

Figure S1. Diagrammatic illustration of the lattice space of $MoSe_2$ crystalline.

Figure S2. Stable configurations of Hg^0 adsorption on $MoSe_2$ crystalline.

Figure S3. Equilibirium capacities (Q_e) of $MoSe_3$ at (a) 75 ^oC and (b) 100 ^oC (inserted with the breakthough curves).

Figure S4. Diagrammatic illustration of the nested tube reactor.

Figure S5. Schematic diagram of the reaction system.

Figure S1. Diagrammatic illustration of the lattice space of $MoSe_2$ crystalline

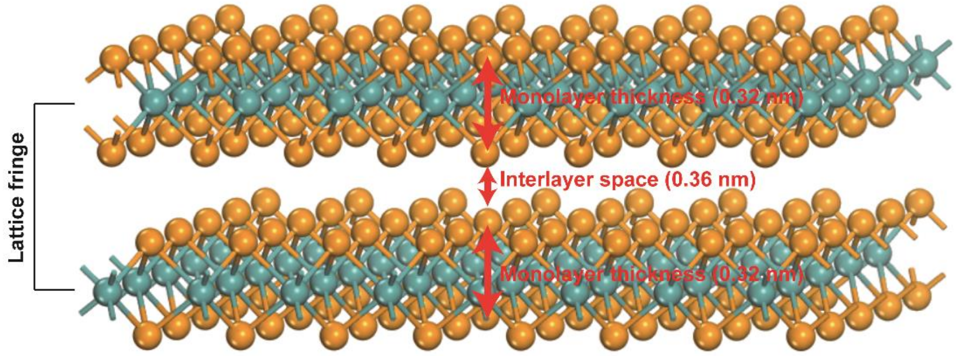

Figure S2. Stable configurations of Hg^0 adsorption on (a) (002) and (b) (100) surfaces of $MoSe_2$ crystalline

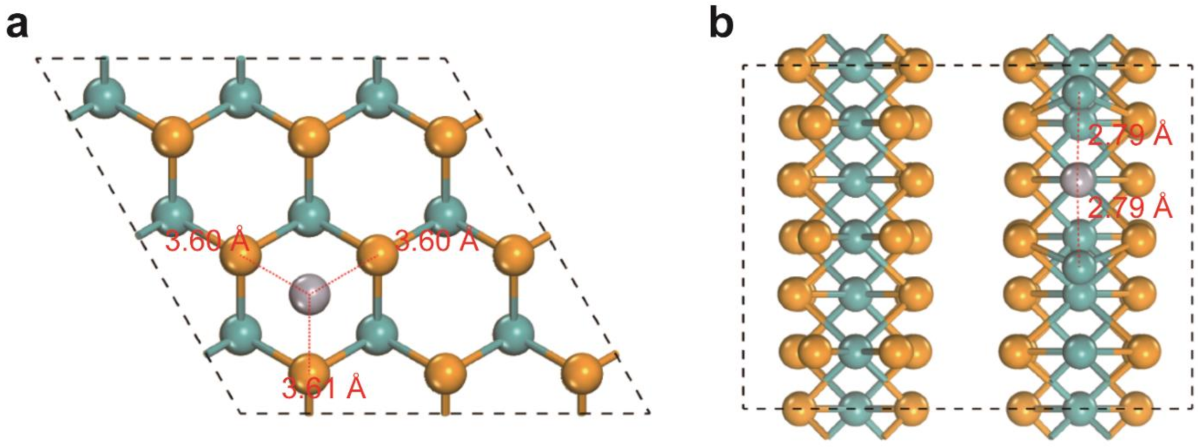

Figure S3. Equilibirium capacities (Q_e) of $MoSe_3$ at (a) 75 ^oC and (b) 100 ^oC (inserted with the breakthough curves).

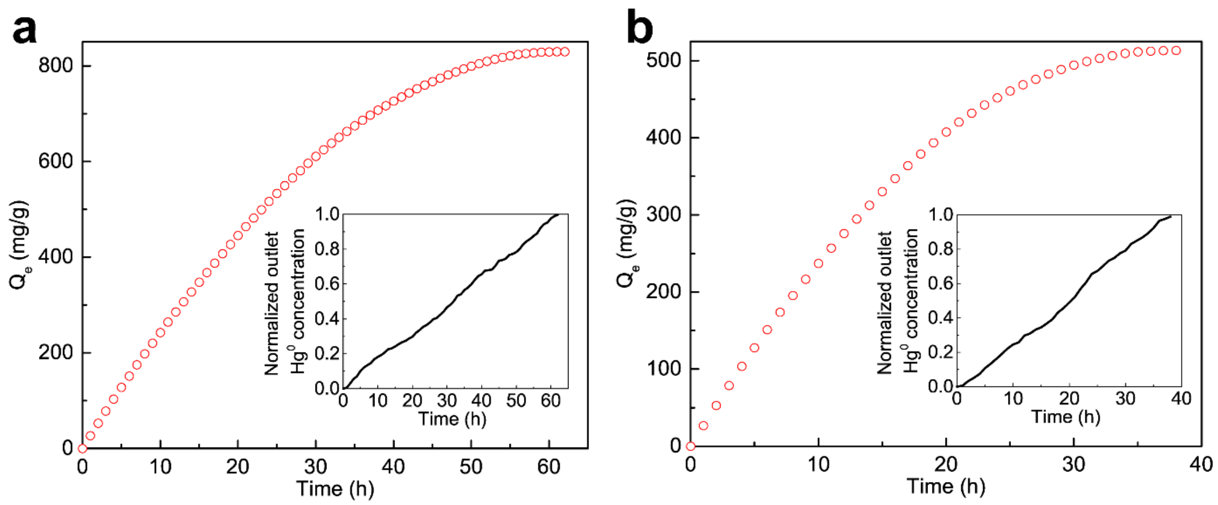

Figure S4. Diagrammatic illustration of the tube reactor

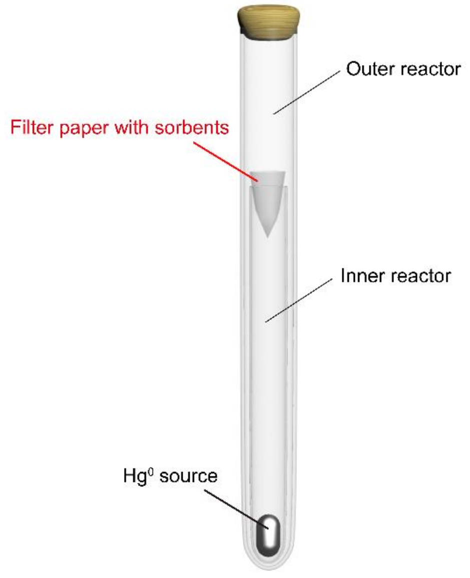

Figure S5. Schematic diagram of the reaction system

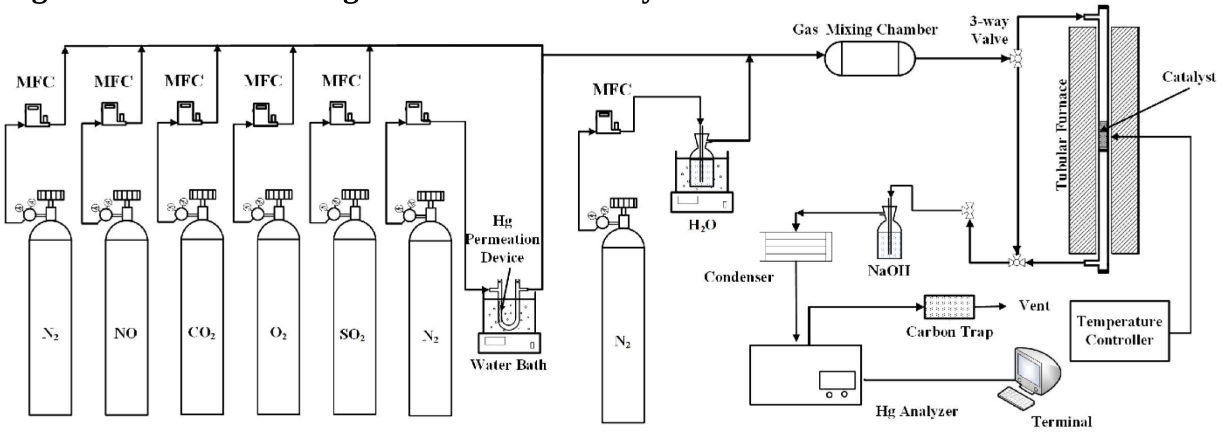

Supplement: Supplementary file 1 — Supplementary [file ADVS-6-1901410-s001.pdf]
